# Arthropod spatial cognition

**DOI:** 10.1007/s10071-020-01446-4

**Published:** 2020-11-10

**Authors:** Sarah Pfeffer, Harald Wolf

**Affiliations:** Institute of Neurobiology, Ulm University, Albert-Einstein-Allee 11, 89081 Ulm, Germany

**Keywords:** Arthropods, Spatial cognition, Navigation, Orientation, Comparative approach

## Abstract

The feats of arthropods, and of the well-studied insects and crustaceans in particular, have fascinated scientists and laymen alike for centuries. Arthropods show a diverse repertoire of cognitive feats, of often unexpected sophistication. Despite their smaller brains and resulting lower neuronal capacity, the cognitive abilities of arthropods are comparable to, or may even exceed, those of vertebrates, depending on the species compared. Miniature brains often provide parsimonious but smart solutions for complex behaviours or ecologically relevant problems. This makes arthropods inspiring subjects for basic research, bionics, and robotics. Investigations of arthropod spatial cognition have originally concentrated on the honeybee, an animal domesticated for several thousand years. Bees are easy to keep and handle, making this species amenable to experimental study. However, there are an estimated 5–10 million arthropod species worldwide, with a broad diversity of lifestyles, ecology, and cognitive abilities. This high diversity provides ample opportunity for comparative analyses. Comparative study, rather than focusing on single model species, is well suited to scrutinise the link between ecological niche, lifestyle, and cognitive competence. It also allows the discovery of general concepts that are transferable between distantly related groups of organisms. With species diversity and a comparative approach in mind, this special issue compiles four review articles and ten original research reports from a spectrum of arthropod species. These contributions range from the well-studied hymenopterans, and ants in particular, to chelicerates and crustaceans. They thus present a broad spectrum of glimpses into current research on arthropod spatial cognition, and together they cogently emphasise the merits of research into arthropod cognitive achievements.

## Introduction

Arthropods are not only highly diverse but many groups, and indeed a few species like krill, are also highly abundant. There are over a million described arthropod species and the total species number is estimated to range between 5 and 10 million. In any case, 80% of all described species are arthropods. These numbers already indicate a correspondingly high ecological significance of arthropods as a group, a view that is substantiated by biomass estimates. About 50% of the total biomass of all animals on earth are estimated to be arthropods (Bar-On et al. 2018). Major reasons for that success include sophisticated behavioural and spatial cognitive feats, and social organisation when present. Examining cognition, especially spatial cognition, would appear important not only for scientific reasons but also for potential applications regarding nature conservation as well as pest control. Humankind strongly depends on arthropods, for instance, with pollination of about 75% of the most important crops being achieved by animals, most of them arthropods (Klein et al. 2007; Potts et al. 2010). In addition, arthropods have been inspiring subjects for bionics and robotics.

The term spatial cognition prominently implies navigational feats, but the term also denotes object and obstacle recognition and associated behavioural actions. Within this framework, the sensory bases for spatial cognition are often also considered. Spatial cognition has been studied in considerable detail in many higher vertebrates, from birds migrating around the globe with pinpoint accuracy to tool-wielding monkeys, apes and, again, birds. What are the merits of studying spatial cognition in arthropods, though?

Honeybees provide part of the answer, not least since they are the one species of all the arthropod creepy-crawlies that have achieved popularity and even certain glamour in the general public. Honeybees, actually a domesticated species like cattle and companion animals of humans since at least 5000 years ago in Egypt (Kritsky 2015), have piqued scientific interest before most other invertebrates (e.g. Turner 1910; Lovell 1910; von Frisch and Rösch 1926; Heran and Wanke 1952). Their cognitive feats are indeed remarkable, including exact navigation to and from foraging places across several kilometres, the learning and association of flower colours, odours and shapes, and even abstract flower qualities like symmetry (Giurfa et al. 1996; Giurfa 2013), and their ability to communicate about feeding places to nest mates back in the hive. Honeybee cognitive performance is considered on par with that of some vertebrates. Although they are not even the smartest arthropods, nor comparable to, say, primates or corvids (Güntürkün and Bugnyar 2016), their abilities are comparable to those of more modest fish (Bshary et al. 2002) or reptile species (Wilkinson and Huber 2012). Although the well-studied honeybee is not among the topics of this special issue, it provides a good example for the merits of arthropod cognition research.

## Small brains

It is remarkable for the honeybee example, and significant for all arthropods, that these creatures achieve their cognitive feats with small brains. The smallest known insects—for instance, wasps of the genus *Megaphragma* or beetles of the genus *Scydosella*—have a body size comparable to that of unicellular organisms like amoebas or paramecia. Their miniature brains compromise about 10,000 cells or less (Polilov 2015), one wasp species possessing only 215 nucleated brain neurons (Polilov 2012). Despite their generally small neuron numbers, these micro-insects exhibit a complete suite of behaviours, including flight, mating or host search. However, the average brain cell number in insects, and more generally in arthropods, is much larger, ranging from about 100,000–1,000,000 cell counts (Meinertzhagen 2010; Polilov 2012). To name a few well-known insect examples, numbers of brain neurons range around 100,000 in the fruit fly *Drosophila* (Zheng et al. 2018), 340,000 in the housefly *Musca* (Strausfeld 1976), and even 1,200,000 in the cockroach *Periplaneta* (Reichert 1993).

By arthropod standards, honeybee workers are thus well endowed with neurons, possessing almost a million nerve cells in their brains (about 810,000; drones have about 1,150,000 (Witthöft 1967; Chittka and Niven 2009), with a brain volume of about 1 cubic millimetre. More than a third of these neurons (about 430,000) reside in the optic lobes, however, processing optic stimuli from the retina and not directly concerned with cognitive processes. The central brain contains about 720,000 neurons, a number comparable to that of other advanced insect species even though on the high end (e.g. Wehner et al. 2007). Almost half of the 810,000 neurons in the worker bee brain, about 340,000 neurons, form the main associative memory network in the mushroom bodies. That network certainly forms a basis for cognitive processes, in particular, associative learning and memory. Although these numbers may appear large on a first glance, they are some five orders of magnitude smaller than those of the human brain, that is, a human brain is equivalent in neuron number to about 100,000 bee brains, with a million nerve cells each equal. The human brain needs much longer axons to connect brain regions due to its size, resulting in a correspondingly larger mass per neuron, as well as significantly more glia cells for wrapping and homeostasis. Thus, the human brain has more than a million times the mass of a honeybee brain.

The relatively small brains of arthropods may help to understand the basic, and perhaps minimum, neuronal requirements for particular cognitive abilities. After all, brain size scales with body mass quite independent of cognitive feats, or some form of intelligence, whatever way it is measured (Chittka and Niven 2009). The absolute neuron counts thus provide little information about the cognitive power of an animal. Factors like cell density, interconnectivity or neural circuit structure play important roles in this context, too. Considering comparable organisational features, larger animals need more neurons than smaller animals due to the larger sizes of their sensory structures and effector organs, particularly muscles (Eberhard and Wcislo 2011). Since the diameters of sensory as well as muscle cells do not vary by more than about an order of magnitude throughout the animal kingdom (Kirschfeld 1984; Wolf 2014), larger animals with correspondingly larger sensory surfaces and muscle volumes require a correspondingly increased number of sensory and motor neurons. This in itself will increase cell numbers and nervous system volumes with body mass. When subtracting brain neuron numbers associated with sensory input and motor output from total neuron numbers, the resulting residual has been considered as a potential measure of processing capacity (Mares et al. 2005; Chittka and Niven 2009), and perhaps cognitive performance, although this remains speculative.

Eventually, arthropods may provide suitable test subjects for such questions. Along these lines, it was one of the incentives for initiating this special issue on arthropod spatial cognition to advocate arthropods for neurobiological study, continuing a line of research that has proven extremely fruitful in the past decades (e.g. Clarac and Pearlstein 2007; Giurfa and Pflüger 2019), including new technical applications (Todd 2013).

## High diversity and comparative research

Another, and perhaps more important, aspect regarding arthropods is their high species and ecological diversity. This diversity can support comparative approaches scrutinising the link between ecological niche, lifestyle, and cognitive competence. After all, cognitive performance has evolved according to the needs of a particular species in its environment and social organisation (Real 1994; Lihoreau et al. 2012; Mettke‐Hofmann 2014). It would appear profitable under this perspective, first, to compare cognitive performances in animal species that are closely related but occupy different ecological niches with different cognitive demands. Here, comparative studies scrutinise the evolution of phenotypic differences with regard to demands in ecological niches and life styles. This approach should be able to illustrate the malleability of cognitive performance and perhaps the limitation of niche exploitation imposed by cognitive limitations. Second, it would appear profitable to study cognitive performances in animal species that are only distantly related but occupy similar ecological niches with similar cognitive demands. Such an approach should be able to elucidate the evolutionary malleability of cognitive performance from a different viewpoint, its mechanisms, and its potential limitations. It should also indicate potentially different strategies to deal with similar cognitive demands. Navigation is a good example here (Bühlmann et al. 2011; Wehner and Wehner 2011; Cheng et al. 2014).

Comparative approaches have more general scientific merits too. General principles in biology have often been recognised when observing similar solutions to a given problem in distantly related organisms. In these cases, the underlying physiological mechanisms will in all likelihood differ in detail since they are not of common origin by descent. That is, they are not homologies inherited from a common ancestor, but rather they are analogies, having been shaped in their similarity by functional requirement. This is well-established and recognised with relative ease for morphological traits ((Losos 2011; prominent examples are the convergent evolution of limb elements in flying pterosaurs, birds and bats (Bell et al. 2011) or the lens eyes of cephalopods and vertebrates (Budelmann 2010)). It is of particular significance for physiological mechanisms and cybernetic control strategies, however, since apparent morphological character sets are usually absent here. The observation of common physiology or control principles in distantly related organisms is thus all the more intriguing. If it cannot be attributed to common origin, it constitutes a convincing case for a physiological mechanism or control strategy shaped by functional necessity, and it immediately begs the question of how general its applicability may be.

The switch from the stance phase to the swing phase in walking is a good example of the generality question. During the stance phase, the leg supports the body mass and moves it forward. The leg reaches a point in some posterior position and extended posture where it cannot be moved further back relative to the body nor can it extend. This is where the leg is usually lifted off the ground in transition to the swing phase that moves the leg forward through the air until it is put on the ground again to start the next stance phase. The switch from stance to swing is contingent on two conditions, and walking will be suspended if not both of them are fulfilled. First, the leg has to be in a posterior and extended position as outlined above, close to limiting further progress in walking. Second, the load has to be taken off that leg by its counterpart, otherwise body mass cannot be supported and the walker will tilt. This rather obvious control principle for stance-swing transition—obvious at least in retrospect—holds for all animals that walk on their legs, including both mammals and insects (Pearson 1993; Aoi et al. 2012), although the last common ancestor of insects and quadruped vertebrates did not have legs for walking. The common ancestor was some urbilaterian with serially arranged appendage pairs used as antennae, or for filter-feeding or swimming (Tabin et al. 1999). The control principle is the obvious result of the functional necessity outlined above. According to its inescapable validity in walking, it has been applied in the construction of technical systems like walking robots (Dürr et al. 2003; Buschmann et al. 2012; Todd 2013).

## Perspectives in arthropod cognition research

With regard to arthropod spatial cognition, a future question may be whether there are arthropods who have an internal representation of the world and their position in this world. Such internal representation, in the sense of Cruse (2003), even if rudimentary, facilitates effective interaction with the world and can be refined by experience. In contrast to motor learning, adjusting an internal representation is more flexible and effective, in particular when it comes to more complex motor planning. The clandestine way salticid spiders approach prey insects (Jackson and Pollard 1996) come to mind when speculating about such internal representation of the outside world. It has also been invoked for honeybee navigation feats (Menzel et al. 2005), although this interpretation remains controversial (Wehner 2009; Hoinville and Wehner 2018; Webb 2019). The recent advances in unravelling the neuronal network assumed to underlie path integration in insect navigation (Webb and Wystrach 2016; Heinze 2017; Honkanen et al. 2019; El Jundi et al. 2019; Warren et al. 2019) may be considered a step in this direction.

Within the arthropods, much like for other animal groups, the potential for comparative studies has not been exploited much. Insects, and in particular the social hymenopterans, bees, bumblebees, wasps and ants, have been studied most intensely, as indicated by the honeybee example above. These animals present excellent opportunities for scrutinising cognitive abilities due to their remarkable achievements in this realm, as well as their abundance and availability, and the relative ease of experimental approaches that may be used. Consider the honeybee again, a species bred as a husbandry animal, where one can observe foragers’ behaviour at experimentally established feeding sites and record communication regarding feeding site location and food quality from the same individuals upon return to the hive (von Frisch 1967). This excellent knowledge base is the reason why bees are represented only by a bumblebee in this issue. It was a second, and indeed a major, incentive for initiating a special issue in arthropod spatial cognition to highlight arthropod groups that have received comparatively little attention, yet may provide rewarding subjects for future studies in this field. Precedence in the issue was thus given to manuscripts on understudied arthropod groups, where possible.

Social hymenopterans are the subjects of most publications in the issue despite the quest for more enigmatic arthropods. After all, they are a diverse group in their own right, providing ample opportunity for (comparative) research even between rather closely related species. Cornelia Bühlmann and colleagues compared the North African desert ant *Cataglyphis fortis* to the Australian desert ant *Melophorus bagoti* (Bühlmann et al. 2011). The two species possess many similarities in ecological niches, lifestyles, and foraging and navigation strategies. They inhabit desert- or steppe-like habitats, both are individual scavengers feeding mainly on arthropod carcasses, body sizes and foraging ranges are similar, and both species are able to employ path integration as well as landmark-based routines for navigation. The typical habitats of the two species differ with regard to visual orientation cues available for navigation, however. *C. fortis* dwells in flat and featureless salt pans, and usually has little chance to employ landmarks, neither during outbound foraging trips nor for homing. By contrast, *M. bagoti* typically lives in steppe-like habitats cluttered with grass tussocks and the occasional shrub or tree. This sparse but distinct vegetation provides ample opportunity for landmark-based navigation. Bühlmann and colleagues demonstrated propensities of the African and Australian desert ants for path integration and landmark-based navigation, respectively, in accord with the species’ typical habitat structure. These results were obtained by subjecting the two ant species to identical experimental procedures, thus eliminating any bias introduced by habitat structure. In essence, the authors provided the ants either with flat terrain or with landmark arrays during either the outbound or the homebound leg of the round trip to a feeding site, in all possible combinations. Despite all their similarities, the two desert ant species placed different emphasis on the two navigation strategies available to them, vector-based versus landmark-based routines. It remains unanswered in this study whether these species-specific propensities are genetically determined or shaped by the individuals’ experiences in their respective environments.

This example also illustrates the fact that a good knowledge base is, of course, a prerequisite for detailed comparative study. This certainly puts our plea for more diverse comparative approaches involving understudied groups and species in perspective. It further suggests using one of the more thoroughly studied species as one object for comparison where possible, the “baseline species” put bluntly, whereas the other subject for comparison would be selected according to the hypothesis at the base of the comparative effort, as outlined above.

The different contributions in this issue show that sophisticated behaviour can be achieved with small and presumably parsimonious brains. Cognitive functions like path integration, landmark guidance, or multiple cue integration can often be deduced to basic neuronal mechanisms. On a cellular or molecular level, the principles of learning and cognition may have profound similarities throughout the animal kingdom (e.g. Pearson 1993; Koch 2017; Schlegel et al. 2017; Dyer et al. 2019). Examining and understanding the fundamental processes in arthropods that are decisive for complex behaviours is a unique opportunity to unravel mechanisms and neuronal circuits that might be used by other (more derived) animals, including humans.

## Overview of special issue contributions

Arthropods demonstrate a remarkable repertoire of behavioural and cognitive feats. Central place foragers are prominent representatives in spatial orientation research, presenting opportunities for navigation research. These species are often social, thus possessing an overwhelming motivation to return home (despite whatever interferences with their homing behavioural biologists care to concoct). To solve the problem of finding their way back home, or to revisit a plentiful feeding site, central place foragers are known to use a plethora of orientation cues and navigation mechanisms. As stated above, honeybees represent the classic and thoroughly investigated insects for navigation, with other hymenopterans, ants in particular, following right after and complementing our knowledge substantially. It should be kept in mind, though, that other arthropods have representatives of social central place foragers as well, or face the problem of returning to nesting sites, webs or shelters after foraging or mating excursions (e.g. crustaceans or spiders (Hoffmann 1985; Wehner 1992), see also below).

Hymenopteran insects, and often ants, make accordingly significant contributions to this special issue. Fleischmann, Groh, and Rössler provide a review on the evidence for magnetoreception in hymenopterans, and discuss the use of the earth’s magnetic field for navigation by desert ants. These animals perform learning walks during their initial outings from the nest to familiarise themselves with the surrounding landscape (Fleischmann et al. 2016). The earth’s magnetic field serves as a reference frame for these early learning walks. Fleischmann et al. (2016) were the first to show the use of the earth’s magnetic field by an insect in a natural navigation context, and in particular with other potential orientation cues available at the same time. With their well-studied navigation performance, desert ants would appear suitable for further unravelling mechanisms and uses of magnetoreception.

The performance of learning walks for acquiring nest-centred panoramic views for orientation is a key stage during early foraging life for many species, especially social insects in visually cluttered environments. This is borne out by the findings of Deeti, Fujii and Cheng. They report that naïve *Melophorus bagoti* ants are able to extrapolate the learned panorama to distances from the nest they had never experienced before. This held true even when the environmental view was partly obstructed during the critical time period of the learning walks. These findings indicate that the comparatively well-studied role of panoramic views in ant homing is far from being exhausted as a research topic.

Visual orientation is also important further on in an insect’s life. Landmarks and the visual panorama are prominent here, providing a scaffold for homing. Islam, Freas and Cheng show that a change in major aspects of the familiar scene (by felling trees and erecting fences for a construction site, in this case) considerably upsets homing performance in *Myrmecia midas* bull ants. This is interpreted as a significant decrease in homing certainty, although other orientation cues were still present, such as celestial star patterns in this nocturnal species. Homing performance recovered fast with further experience, however, with regard to both correct choice of homing direction and straightness of homing path.

Visual perception is not only employed for navigating towards a goal, for example, the nest of a homing animal but also for avoiding objects obstructing a chosen path. Baird studied how bumblebees avoid an object in their flight path. The results demonstrate similar visual avoidance of the object under both dim and bright light conditions. Although this agrees with bumblebees’ natural foraging behaviour in bright daylight as well as during dusk, the result is unexpected since under dim light visual perception should be compromised (Warrant and McIntyre 1992). The only significant difference was a slower flight speed in dim light, perhaps allowing for longer integration time for object and speed recognition.

There is, of course, more to orientation and navigation than visual cues. Path integration is another mechanism used by able navigators, particularly important when other cues are unavailable or unreliable. Many arthropods have been shown to use path integration, at least in certain circumstances (Cheng 2006). Freas, Congdon, Plowes and Spetch scrutinised the use of path integration vectors in foraging harvest ants, *Veromessor pergandei*, when returning home. These animals exploit food patches at distances of some 3–40 m from their nest. The patches are accessed via a column trail marked by pheromones. Around the food patches, the individual foragers use path integration to fan out, retrieve food items, and return to the head of the column. They follow the column trail back to the nest, guided not only by pheromones but also by a column path integration vector. The authors showed that it is the presence of the pheromones, rather than visual cues around the column head, that triggers homing along the column by activating the column vector, safely leading the ants back to the nest. These ants thus maintain a memory of two vectors and the corresponding locations, and can switch between them according to the (pheromone) situation.

The above navigation mechanisms are efficient and reliable enough to guide the animals home safely after foraging excursions or other outings, or to a familiar feeding site, at least under normal circumstances. Navigation errors do occur, however, particularly when animals have been transported passively, for instance by a wind gust or by a predator they manage to escape from after some struggle. If the animals fail to find the nest after having run off their home vector, they initiate a systematic search behaviour (Wehner and Srinivasan 1981). The two of us, Pfeffer and Wolf, together with Wahl, compared this search behaviour in two closely related North African desert ants. The silver ant, *Cataglyphis bombycina*, is a specialist inhabiting Saharan sand dune areas, while *Cataglyphis fortis* dwells in salt pan habitats with level and hard-baked clay floor. The silver ant clearly has the more difficult, yielding dune sand substrate for walking, and this ant had been shown previously to exhibit a distinct walking behaviour probably related to locomotion through fine sand. Stride frequencies are high, the ants possess an unusually strict tripod gait, they exhibit pronounced aerial phases (they “gallop”) and extremely brief ground contact times of their tarsi. The presumably more difficult locomotion on dune sand at high speed would be expected to compromise path integration. Nonetheless, the accuracy of nest searches turned out to be similar in the two ant species, and it was even independent of the actual substrates the two ant species were required to run on in the experiments. What differed significantly, however, was the spread of the nest searches. The silver ant spreads its search across a larger area, presumably to compensate for an increased uncertainty of the path integrator in its normal sand dune habitat.

A good navigator should be able to combine more than one cue when meandering through the environment and to find a familiar food source, for example. This should increase both navigation accuracy and certainty, and safeguard against changing environmental conditions (such as the felled trees used by Islam and colleagues to study the effects of panorama changes). De Agrò, Oberhauser, Loconsole, Galli, Dal Cin, Moretto and Regolin have studied such cue combination in the common garden ant, *Lasius niger*. In Y-maze experiments, they combined an odour cue with a colour of the maze wall that led the ants to a food source in a specific odour-colour combination. The animals were able to integrate the two cues that were apparently redundant in training, and to reliably find the food even when, for example, the odour was made uninformative by labelling both arms of the Y-maze with the odour. In this test situation, ants could successfully predict the location of the expected reward by following visual and chemical cues, which demonstrated that they are able to extract and correctly combine contextual information. Interestingly, ants memorised the cue combination even before its predictive meaning was assessed (to some extent even during the first encounter). This outcome is in line with previous results gained from multi-modal learning experiments (see below).

The thorough review by Buehlmann, Mangan and Graham expands our view of multimodal cue interaction in navigation across the insect taxon. Animals have a broad spectrum of sensory inputs at their disposal when moving through their world, from personal idiothetic signals to local cues like landmarks, wind or odours, and to global cues in the shape of the earth’s magnetic field or skylight compasses. The authors argue that animal species are likely to use all those cues that may significantly contribute to navigation performance in situations the animals may encounter in their lives. This will increase navigation performance as noted above, and robustness against perturbations or the occasional lack of particular cues. It is thus no surprise that multimodal cue use is observed in most aspects of navigation, at least when the respective scrutiny is applied, and a species is not used as a model organism for one particular aspect in its life. Buehlmann and colleagues take us through all relevant facets of multimodality in navigation by insects.

The above studies on ants and bumblebees have dealt with cognitive performance of the individual, rather than with social communication about spatial matters like feeding site locations. Social information transfer is well established for honeybees, in the form of round and waggle dances, when returning foragers inform their nest mates about the location and yield of feeding sites (von Frisch and Lindauer 1956) and provide forage samples to further inform about the food odour. Ants transfer spatial information to nest mates, too, although in somewhat less sophisticated ways. They typically use pheromones to mark trails to food sources, or sometimes more exotic modes of nest mate recruitment like tandem running. In tandem running, a teacher ant leads the way for a student to follow and learn the path to a site the teacher has previously examined and found worthy to recruit nest mates (Franks and Richardson 2006). Reznikova reviews work on foraging styles and information transfer in ant colonies of the *Formica rufa* group. Whereas the desert ants described above are solitary foragers that navigate through often lengthy foraging trips all by their own, leader scouting for *Formica rufa* ants is a social endeavour. Scouts reconnoitre the nest environs for suitable food sources and communicate back to colony foragers for further exploitation. There is a division of labour, thus, not only regarding foraging and scouting but also the cognitive capacities necessary for the respective tasks. A special merit of Reznikova’s contribution is the fact that she reviews work that has often been published only in Russian so far, now making it available to the broader scientific community.

There is no rule without exception in biology, and dung beetles provide a good example for that, as reported by Dacke, El Jundi, Gagnon, Yilmaz, Byrne and Baird. The beetles do not use landmarks for homing to their burrow with a dung hoard established for provisioning the larvae. Oblivious of the above line of argument for multimodal cue integration in navigation, *Scarabaeus galenus* relies exclusively on path integration to add further dung pellets to its burrow from nearby antelope droppings. The beetle does this despite ample availability of landmarks in its biotope. These beetles are actually special in several ways. For one, the mere fact that they have a burrow they repeatedly return to for adding (small antelope) dung pellets is unusual. Most scarabaeid dung beetles slice off adequate pieces from large, soft droppings like those of elephants, to shape into a sizeable ball they roll away. Further, the beetles home to their burrow running backwards, holding the pellets between their hind legs. One may expect further news on these dung beetles considering their score of understudied feats.

The contributions to follow are of particular interest insofar as they do not deal with ants or bees, not even with insects, but with other, relatively little studied arthropods. Following a taxonomic path, hermit crabs are next. According to current systematics both insects and crabs are crustaceans, after all. Krieger, Hörning and Laidre provided artificial, 3D printed snail shells to hermit crabs to slip into and carry around as shelters. In this way, they were able to offer the crabs a choice of shells, including those they prefer according to suitable size, interior smoothness, and correct handedness. Only a non-preferred shell enabled the animals to escape from an enclosure; however, A box the crabs were confined to during the shell choice experiment. The non-preferred shells had outer spines that were too large to fit through an escape opening but were somewhat uncomfortable because they were either too small, had small inner spines, or had left- instead of the usual right-handed turns. The terrestrial *Coenobita compressus* crabs are keen to be around conspecifics and have a high motivation to escape loneliness in their enclosure. Indeed, a majority of the animals were willing to forgo in their choices the preferred shell type in favour of the non-preferred shell that allowed them to escape confinement. Although the concern remains from this pilot study, whether the crabs solved the escape problem by an awareness of the underlying spatial problem or by trial and error, the current experimental paradigm instigates further investigations of hermit crabs’ spatial and social cognition capacities.

The following three studies deal with chelicerates, an enigmatic group not only with regard to navigation research. Ortega-Escobar reviews homing in aranaeans and amblypygids, two Chelicerata taxa that have been studied to some extent. Many aranaean spiders live in burrows or silk tubes they leave to capture prey and quickly return to for shelter after a successful strike. The same holds for the amblypygids, or whip spiders, although they typically wander farther from their shelter during prey capture excursions. Ortega-Escobar reviews and discusses sensory bases and available orientation cues for homing, which appears to be primarily based on path integration in most of the investigated species.

The study by Casto, Wiegmann, Coppola, Nardi, Hebets and Bingman examines navigation in whip spiders and thus, in a way, extends the material reviewed by Ortega-Escobar. Different from most other studies on navigation in this issue, and actually from most studies concerned with homing animals, Casto and colleagues examined vertical-surface navigation. The study by Islam, Freas and Cheng noted above is one of the rare exceptions. Although vertical-surface-navigation was not specifically examined in this study, their tested animals, bull ants, generally navigate on more or less vertical tree trunks while foraging. This is just what many whip spiders do, including *Paraphrynus laevifrons*. Living on trees, they have to cope with vertical surfaces most of their lives. The authors investigated the whip spider’s ability to return to a home shelter in the presence of numerous other refuge sites, and they examined the role of sensory cues emanating from a previously used shelter in competition with shelter position. The animals exhibited robust homing behaviour and navigation errors, where observed, did not differ between the horizontal, vertical, or diagonal planes. Cues from previously used shelters, probably chemical signals deposited by the animals, appeared to take precedence over positional information.

Last but most certainly not least, Prévost and Stemme present a pilot study on homing in scorpions, and they discuss their results against a review of the current status of scorpion navigation research. A major goal of the authors was to test a laboratory setup for studying scorpion homing and general navigation abilities. They further scrutinised the roles of vision and chemosensation for homing, and concluded that the animals most probably perform path integration when leaving their shelter to roam the surroundings and later return home.
